# Impact of maternal employment on child health and nutrition: Evidence from Nepal

**DOI:** 10.1371/journal.pone.0355481

**Published:** 2026-08-13

**Authors:** Khushbu Mishra, Olga Kondratjeva, Abdoul G. Sam

**Affiliations:** 1 Department of Economics, Stetson University, Deland, Florida, United States of America; 2 Washington State Employment Security Department, Olympia, Washington, United States of America; 3 Department of Agricultural, Environmental and Development Economics, The Ohio State University, Columbus, Ohio, United States of America; UF: University of Florida, UNITED STATES OF AMERICA

## Abstract

The high prevalence of child undernutrition has detrimental consequences, including lower educational attainment and reduced lifetime earnings, thereby trapping many families in intergenerational poverty. Therefore, identifying the factors that influence child nutrition is an important policy issue. Using nationally representative data from Nepal, we investigate whether maternal employment improves child nutrition and growth, as measured by height-for-age, weight-for-age, and weight-for-height among children under five years of age. Applying coarsened exact matching and an instrumental variable approach, we find that maternal employment has no statistically significant effect on child nutritional outcomes. Maternal employment in Nepal may not improve child nutrition because income gains from predominantly informal work are offset by reduced time available for childcare and feeding. In addition, limited female bargaining power and control over household resources in patriarchal and gerontocratic households may weaken the link between maternal earnings and child health outcomes.

## Introduction

Women juggle multiple roles in their households. While traditionally they primarily played the role of a caregiver to their children and other household members, with increasing emphasis on gender parity, women’s participation in the labor force is increasing. As a result, women are becoming overburdened with the added role of providing for their families. In particular, the share of women in the labor market is increasing in developing countries [1]. Mothers’ employment can take time away from their children which may lead to sub-optimal childcare. This in turn can translate into negative impacts on children’s health and nutrition [2–5]. Alternatively, employed mothers, through added earnings, have the capacity to invest more in children’s health and nutrition leading to positive child outcomes [6]. Which of these arguments hold? In this article, we investigate the impact of maternal employment on children’s health. In particular, we examine the impacts on children’s health using underweight (weight-for-age), stunting (height-for-age), and wasting (weight-for-height) outcomes for children under the age of five.

The relationship between maternal employment and child health has been examined in the literature before. One of the first papers to examine this relationship was Lamontagne, Engle, and Zeitlin [7]. Using data from low-income urban communities in Nicaragua, the authors find that children of employed mothers had better outcomes in weight/height than those of unemployed mothers, with earnings proposed as the primary mechanism. Similarly, using a welfare enhancing work program in rural India, Diiro, Sam, and Kraybill [8] find that children in the lower tail of the height-for-age distribution benefit the most from maternal labor market participation. In contrast, Gennetian et al. [9] find a modest adverse effect of maternal employment on children’s health of low-income, elementary-school-aged children using data from a 1990s welfare-to-work program in the US. Furthermore, Liu et al. [10] use the 2000 wave of the US National Longitudinal Survey of Youth 79 and find that children of full-time working mothers have a higher likelihood of being overweight when measured by body mass index (BMI). Likewise, Maddah et al. [11] find that children of employed mothers were more likely to be underweight, stunted, and wasted compared to those of unemployed mothers in Rasht City in Iran. Additionally, recent studies have found children whose mothers were employed had higher incidence of wasting and being underweight and lower height-for-age Z-score [4,5]. While these positive and negative impacts of maternal employment on child health may seem contradictory, the former is attributed to increased earnings and the latter to mother’s time away from childcare and lack of breastfeeding. In this regard, Baker and Milligan [12] investigate the impact of increased maternity leave mandates in Canada on child health and find no effect for most indicators.

While these studies are useful in understanding the relationship between maternal employment and child health, most of them study countries in Africa and the Americas. To date, there are no nationally representative studies that investigate the impact of maternal employment on child health in Nepal and very few that explore the broader South Asian context. Therefore, we propose to extend the existing literature concerning the impact of maternal employment on child health with the aim of shedding light on the potential implications of this relationship in Nepal and South Asia. Hence, by using a nationally representative dataset from Nepal, we contribute to the limited South Asian literature on this topic. Second, this study seeks to provide deeper insights into women’s labor force participation and child health by using robust econometric techniques like the coarsened exact matching and instrumental variables approaches.

Using two econometric techniques and several specifications, we find that maternal employment has no impact on child nutrition, as measured by stunting, underweight, and wasting, on average. Our results lie between the two perspectives discussed above such that one set of studies proposes that maternal employment is detrimental to child nutrition and another proposes that it improves child nutrition [8,13]. These findings are consistent with the Canadian study on maternal leave with null effects on child health [12]. Overall, the results are context-specific to Nepal because mothers (and females broadly) are engaged in informal work which suggests that modest income gains may be offset by reduced time for childcare and feeding [14]. Additionally, households are largely patriarchal and gerontocratic, limiting women’s and younger mothers’ bargaining power and control over resources, which can weaken the link between maternal earnings and child health outcomes [15]. Our study contributes to policy discussions on not only on increasing women’s income opportunities but also on enhancing their decision-making power and access to formal childcare support.

## Background and data

This study investigates the impact of maternal employment on child health through child nutrition indicators in Nepal. At the national level, the NLSS defines “currently active” as “either employed for at least one hour during the previous seven days, or has a job attachment if temporarily absent from work, or is available to work if work could be found”. Similarly, it defines “employed” as “either employed for at least one hour during the previous seven days, or has a job attachment if temporarily absent from work” [16]. Per this definition, the female labor force participation rate is 79.4%, and the proportion of employed females is 78.3% with roughly 40% of them working more than 40 hours per the 2010/11 NLSS. The majority of employed females are engaged in self-agriculture (67.7%). Similarly, the survey collected information on the key indicators for monitoring the nutritional status of children under the age of five: stunting (height-for-age), underweight (weight-for-age), and wasting (weight-for-height). To calculate these, the survey collected information on children’s height, weight, and age in months. In particular, a child under five is defined as stunted or severely stunted if she falls below minus two or minus three standard deviations from the median height-for-age World Health Organization (WHO) reference population, respectively. At the national level, 42% of children under five are stunted and 15% are severely stunted [16].

Stunting is more prevalent in the Mountain region compared to Hill and Tarai regions. Likewise, underweight and severely underweight children are defined as the proportion of children under five that fall below minus two and minus three standard deviations from the median weight-for-age WHO reference population, respectively. The proportions in these categories are 31% and 8%, respectively. The prevalence of underweight children is roughly twice as high in rural areas as in urban areas. A child under five is defined as wasted or severely wasted if she falls below minus two and minus three standard deviations, respectively, from the median weight-for-height WHO reference population. About 14% of children in Nepal are wasted and 3% are severely wasted. The Tarai region has the highest prevalence of wasting among children compared to Hill and Mountain regions. All three indicators are worse for households in the lowest wealth quintile [16].

### Data source

The data for this study come from two waves of the Nepal Living Standards Survey (NLSS), 2003/04 and 2010/11, collected by Nepal’s CBS and designed to track changes in living standards and social indicators of the Nepali population. These surveys follow the methodology of the Living Standard Measurement Survey designed by the World Bank for over 50 countries. The NLSS is a nationally representative household survey that collects comprehensive household-level information on demographic composition, dwelling characteristics, geographic location, household income and consumption, borrowing behaviors, and agricultural and business activities. In addition, it collects individual-level data on household members’ age, ethnicity, educational attainment, and employment status, including, of relevance for this study, maternal employment and child health indicators.

The NLSS 2010/11 is the primary data used in the study. The survey includes a cross-sectional and a panel component, where the cross-sectional component reflects a nationally representative sample of households and the panel component includes households that were surveyed in the previous waves of the NLSS. The total sample size in 2010/11 includes 7,020 households—a cross-sectional sample of 5,988 households (including 28,670 individuals) and a panel component of 1,032 households (including 5,416 individuals) [16]. The NLSS 2003/04 is used as a supplementary dataset to create an instrumental variable for the analysis. The sample selection for the 2003/04 survey follows the same sampling method as the 2010/11 survey, and includes a panel and a cross-sectional component, with 4,008 and 1,232 households for each component, respectively.

Since we investigate the impact of maternal employment on child health, the unit of analysis for this study is at the child level. The construction of our sample followed three steps. First, we used cross-sectional data from the 2010/11 survey, limiting the sample to children who were 59 months or younger at the time of the survey (*n* = 2,846). After excluding observations with missing information and conducting data cleaning, the cross-sectional sample for 2010/11 consists of a total of 2,431 children (from 1,845 households). Of these 2,431 observations, 4 observations were missing data required for summary statistics, resulting in 2,427 observations in Table 2. Second, we used detailed survey information on the composition of a household to match children’s data to their mothers’ data. Third, as described below, we used the matching techniques to merge the 2010/11 sample with the 2003/04 survey wave. Following the matching process, the final analytical sample dropped to 2,410 children (from 1,836 households) who were 59 months or less in 2010/11. Of these, 32 were missing observations on control variables related to distance from infrastructure developments, leaving a final 2,378 observations for Tables 4-S10 (S1 Appendix).

### Key variables

The key measures come from the 2010/11 wave. Our key variables of interest are maternal employment status and anthropometric measures that identify early childhood growth among children under the age of five. In the main model, maternal employment is defined as 1 if the mother worked during the past 7 days and 0 otherwise; this work can be either wage employment or self-employment. Additionally, we use an alternative definition of maternal employment, which is defined as whether a mother was engaged in either wage employment or self-employment in the past 12 months. For anthropometric characteristics, we use the three standard measures—height-for-age, weight-for-age, and weight-for-height—measured as continuous variables that represent calculated anthropometric z-scores based on the 2006 WHO growth standards [17]. A higher height-for-age z-score (HAZ) corresponds to a greater child height relative to age, and a lower HAZ may signify a degree of stunting. A higher weight-for-age z-score (WAZ) corresponds to a relatively higher weight whereas a lower WAZ may describe some degree of underweight. Lastly, a higher weight-for-height z-score (WHZ) may reflect relatively higher weight or relatively lower height, and a lower WHZ may describe some level of wasting. Per previous WHO research, we use pre-specified cut-offs to exclude extreme values that are likely the result of measurement or data entry errors: a child is excluded if their height-for-age or weight-for-age score were below −6 or over +6, or their weight-for-height score was below −5 or over +5 [18].

Other than outcomes and determinant variables, we surveyed the literature to identify key covariates widely used in the context of maternal employment and its impact on child growth and nutritional status [2,8,19–21]. Based on the literature review, we include the following covariates in the study: child characteristics (gender, age, age squared, birth order, number of vaccinations received, breastfeeding status), mother characteristics (age and education), household characteristics (ethnicity and gender of a household head, whether father engages in paid work, household members aged 6–15 and 65 years and over, number of other working household members, number of female household members excluding the child’s mother, income quintile, household index for hygiene and sanitation, household index for female empowerment, whether a household is located in a rural area, and household’s ecological zone), infrastructure development (distance (in kilometers) to a healthcare facility, primary school, paved road, market center, and bank) and district characteristics (prenatal care utilization). The household-level index for hygiene and sanitation captures the average of six indicators: (i) whether the household’s drinking water comes from a piped water supply, (ii) whether a household is connected to a sanitary system for liquid wastes, (iii) whether garbage is disposed via a garbage truck or a private collector, (iv) whether the household has access to a toilet, (v) whether the household’s cooking stove emits no fumes indoors, and (vi) whether concentration of iodine was over 15 ppm, in line with WHO standards. The household-level index for female empowerment measures the degree to which a female household head or the spouse of a male household head has substantial involvement in the following household decisions: (i) making a decision to obtain health care for self, (ii) obtaining health care for children, (iii) spending on food, and (iv) spending on major household items. The household-level index for female empowerment is the average of the four indicators.

The key variables in the study are drawn from the 2010/11 survey wave. In addition, we include a measure of lagged community-level female employment from the 2003/04 survey as an instrument in our instrumental variable regression analysis. This variable captures the proportion of working females in the community who worked in the past seven days (see the Empirical Model Section for details). Table 1 describes the variables used in the study and their definitions.

**Table 1:** Description of variables.

Variable	Description
*Outcome variables (2010/11)*	
Height-for-age	Height-for-age z-scores (HAZ) using the 2006 WHO growth standards is derived based on information on a child’s age, gender, length/height, and the mode of data recording (standing vs. lying).
Weight-for-age	Weight-for-age z-scores using the 2006 WHO growth standards (WAZ) are derived based on information on a child’s age, gender, and weight.
Weight-for-height	Weight-for-height z-scores (WHZ) using the 2006 WHO growth standards is derived based on information on a child’s age, gender, length/height, weight, and the mode of data recording (standing vs. lying).
*Covariates (2010/11)*	
Mother is employed	Indicates whether a mother conducted a job in the past 7 days or the past 12 months. Work may include wage employment (in agriculture and outside of agriculture) and self-employment (in agriculture and outside of agriculture).
Child is female	Indicates whether a child is female.
Child age	Child’s age in months.
Child birth order	Child’s birth order to a mother.
Ratio of vaccines	The number of vaccinations, including BCG (against tuberculosis), DPT (against diphtheria, pertussis, and tetanus), Polio, Measles, Hp-B (against Hepatitis B) divided by the total number of vaccines that could be received.
Breastfeeding status	Indicates whether a child was exclusively breastfed until 6 months of age (children aged 6 months or older), or whether a child has been exclusively breastfed until now (children under 6 months).
Mother’s age	Mother’s age in years.
Mother’s education	Indicates mother’s level of educational attainment (never attended school, grades 0–6, grades 7–10, School Leaving Certificate or above).
Mother’s birthplace	Indicates whether the mother lives in the district where she was born.
Father engages in paid work	Indicates whether father lives in the same household and engaged in wage employment in the past 7 days.
Household head is female	Household head is female.
Household head ethnicity	Household head’s ethnicity (Chetri, Brahman, Newar, Other).
Household size	The number of household members.
Household members by age groups	The number of household members aged 6–15 years and 65 years or older.
Number of other working members	Number of household members engaged in any work in the past 7 days (wage employment or self-employment), excluding mother and father.
Number of adult females in household	Number of adult women (16 years old and over) in the household, excluding mother.
Index for hygiene and sanitation	An average of six indicators: (i) whether household’s drinking water comes from piped water supply; (ii) whether a household is connected to a sanitary system for liquid wastes; (iii) whether garbage is disposed via garbage truck and private collector; (iv) whether household has access to toilet; (v) whether stove for cooking emits no fumes within households; and (vi) whether concentration of iodine was over 15 ppm, in line with WHO standards.
Index for female empowerment	An average of four indicators for which the spouse of a male household head or a female household head was heavily involved in: (i) making a decision to obtain health care for self; (ii) obtaining health care for children; (iii) spending on food; (iv) spending on major household items.
Value of durable household assets	Value of durable household assets acquired more than 12 months earlier (categorized as no assets, and positive assets divided into terciles).
Access to healthcare facility	Distance to any healthcare facility (kilometers).
Access to primary school	Distance to primary school (kilometers).
Access to paved road	Distance to paved road (kilometers).
Access to market	Distance to market (kilometers).
Access to bank	Distance to bank (kilometers).
Rural	Indicates whether a household is in a rural area.
Ecological region	Ecological region (Mountain, Hill, Tarai).
District-level use of prenatal care	Use of prenatal care at health care facility during the last pregnancy at a district level.
Total household income	Household income from any agricultural and non-agricultural sources (divided into 5 quintiles).
*Instrumental variable (2010/11)*	
Community female employment	The number of females in a community conducting work in the past 12 months as a proportion of all females in a community. Community is defined in terms of a district. For each observation, the calculation of the proportion excludes the values of the specific observation.
*Instrumental variable (2003/04)*	
Lagged community female employment	The number of females in a community conducting work in the past 7 days as a proportion of all females in a community. Community is defined in terms of a Primary Sampling Unit (PSU).

Notes: Variables are based on 2010/11 survey unless noted otherwise.

### Descriptive statistics

We present summary statistics of the key characteristics of the study sample, including t-test mean comparisons of sample means by maternal employment status, in Table 2. In the sample, 49.5% of mothers were employed in the past seven days and 74.7% of mothers were employed in the past 12 months.

**Table 2:** Summary statistics (2010/11 NLSS).

Characteristic	Total	Not employed	Employed	Diff.
Mother is employed (past 7 days)	0.495			
Mother is employed (past 12 months)	0.747			
*Child characteristics*				
Child is female	0.48	0.486	0.474	ns
Child age (months)	30.3 (16.89)	28.3 (17.20)	32.285 (16.31)	***
Child birth order	2.61 (1.72)	2.418 (1.59)	2.804 (1.83)	***
Ratio of vaccines	0.81 (0.263)	0.781 (0.279)	0.839 (0.24)	
Vaccines are missing	0.178	0.186 (0.39)	0.170 (0.38)	
Did breastfeed	0.737	0.764	0.709	ns
*Mother characteristics*				
Mother’s age (years)	27.5 (6.16)	26.720 (5.90)	28.373 (6.31)	***
Mother’s education (grades 0–6)	0.217	0.213	0.222	*
Mother’s education (grades 7–10)	0.161	0.179	0.144	***
Mother’s education (SLC+)	0.132	0.137	0.127	***
Mother’s education (no school)	0.49	0.472	0.507	***
Mother’s birthplace	0.647	0.590	0.704	
*Household characteristics*				
Father is engaged in wage employment	0.256	0.270	0.242	
HH head is female	0.225	0.236	0.214	ns
HH head ethnicity (Chetri)	0.167	0.136	0.199	**
HH head ethnicity (Brahman)	0.115	0.122	0.108	ns
HH head ethnicity (Newar)	0.046	0.031	0.061	ns
HH head ethnicity (Other)	0.672	0.710	0.633	***
No. of people in HH	6.579 (2.89)	6.679 (2.99)	6.465 (2.77)	**
No. of children 0–5 years old	1.821 (0.95)	1.904 (1.00)	1.732 (0.89)	**
No. of HH members 6–15 years old	1.309 (1.30)	1.212 (1.27)	1.406 (1.32)	***
No. of HH members over 65 years old	0.282 (0.53)	0.288 (0.54)	0.276 (0.52)	ns
No. of other working members	1.122 (1.49)	0.906 (1.32)	1.342 (1.61)	
No. of adult females in HH	0.973 (1.08)	1.021 (1.09)	0.921 (1.06)	
Index for hygiene and sanitation	0.355 (0.29)	0.358 (0.31)	0.353 (0.27)	
Index for female empowerment	0.863 (0.73)	0.890 (0.73)	0.838 (0.72)	
No HH assets	0.303	0.172	0.266	
HH in 1st asset tercile	0.276	0.274	0.326	
HH in 2nd asset tercile	0.206	0.313	0.239	
HH in 3rd asset tercile	0.218	0.241	0.170	
Distance to healthcare facility	2.50 (3.09)	2.148 (2.78)	2.855 (3.35)	
Distance to primary school	0.861 (0.93)	0.92 (0.97)	0.80 (0.88)	***
Distance to paved road	16.4 (29.9)	21.0 (33.7)	11.8 (24.8)	***
Distance to market	9.53 (11.0)	10.4 (11.4)	8.68 (10.6)	***
Distance to bank	14.4 (16.7)	16.6 (18.2)	12.2 (14.7)	***
Rural	0.761	0.725	0.798	***
Ecological zone: Mountain	0.079	0.033	0.126	***
Ecological zone: Hill	0.482	0.412	0.554	***
Ecological zone: Tarai	0.439	0.555	0.320	***
District-level use of prenatal care	0.133	0.137 (0.05)	0.129 (0.05)	
Total income quintile: 1st	0.2	0.194	0.205	ns
Total income quintile: 2nd	0.2	0.189	0.212	***
Total income quintile: 3rd	0.2	0.191	0.210	**
Total income quintile: 4th	0.2	0.203	0.195	***
Total income quintile: 5th	0.2	0.222	0.178	***
*Dependent variables*				
HAZ	−1.52 (1.56)	−1.375 (1.62)	−1.672 (1.47)	***
WAZ	−1.36 (1.17)	−1.335 (1.19)	−1.383 (1.14)	***
WHZ	−0.71 (1.22)	−0.803 (1.24)	−0.614 (1.20)	***
*Instrumental variables*				
Community female employment (2010)	0.700	0.657	0.745	*
Lagged community female employment (2003)	0.700	0.692	0.699	*
Observations (children)	2,427	1,225	1,202	

Notes: Statistical significance: ***p < .01, **p < .05, *p < .1. HH = household; SLC = School Leaving Certificate. Standard deviations in parentheses where reported.

For remaining covariates, a slight majority of children are male (52%), with an average age of 30.3 months and an average birth order of 2.6. Most children (73.7%) have been breastfed and received most of their vaccinations. The average age of mothers is 27.5 years. A high proportion of mothers either have no schooling (49%) or attended only primary school (0–6 grades, 21.7%). About one-quarter of fathers (25.6%) engaged in wage employment. Nearly 23% of households have female household heads. Over 16% identify as Chetri, 11.5% identify as Brahmans, and 4.6% are Newars; the remaining 67.2% belong to other ethnic groups. The average number of other working adults in a household was 1.2, and the average number of adult female household members, excluding the child’s mother, was 1. On average, households scored relatively low on the hygiene and sanitation index (0.36) and high on female empowerment (0.86). Approximately a quarter of households did not have durable assets. Regarding healthcare facility, the average distance was 2.5 kilometers. In terms of geographic location, three-fourths of households are located in rural areas, and most households live in either Hill or Tarai, with just 7.9% living in the Mountain region.

Overall, children whose mothers work and those whose mothers do not work are different from each other. Those with working mothers are, on average, older, receive a higher rate of vaccines, are of a higher birth order, and are less likely to be breastfed. Working mothers are, on average, older and with lower levels of education. Children with working mothers also tend to live in smaller households (though are more likely to have older children living in a household), are more likely to have fathers who do not work for wages, and tend to score lower on female empowerment. Finally, children with working mothers also report living in poorer households, are more likely to be rural, and are more likely to come from Mountain or Hill zones.

For the outcome variables, the mean height-for-age z-score (HAZ) was −1.52, the mean weight-for-age z-score (WAZ) was −1.36, and the mean weight-for-height z-score (WHZ) was −0.71. These measures differ significantly between children of employed and unemployed mothers. Children whose mothers were employed had lower HAZ scores, slightly lower WAZ scores, and higher WHZ scores than children whose mothers were unemployed.

## Empirical model

We use the following empirical model to examine the relationship between maternal employment and child health:


$$\begin{array}{c} Y_{ijkl}=\beta_{0}+\alpha M_{ijkl}+\beta_{1}C_{ijkl}+\beta_{2}X_{jkl}+\beta_{3}H_{kl}+\beta_{4}G_{l}+\varepsilon_{ijkl}, \end{array}$$ (1)

where *i*, *j*, *k*, and *l* index for child, mother, household, and district, respectively. Here α is the parameter of interest that measures the impact of maternal employment ($M_{jkl}$) on outcome variables ($Y_{ijkl}$) defined by three measurements of child growth and nutritional status: HAZ that assesses the level of stunting, WAZ that assesses the level of underweight, and WHZ that assesses the level of wasting among children under the age of five. Here, $Y_{ijkl}$ is a continuous variable and we run separate regressions for each of the outcome variables. $M_{jkl}$ is a binary variable equal to one if a mother worked either in wage employment or self-employment in the past seven days, and zero otherwise. In an alternative specification, the binary variable indicates if a mother worked either in wage employment or self-employment in the past 12 months. Control variables account for child ($C_{ijkl}$), mother ($X_{jkl}$), household ($H_{kl}$), and geographic ($G_{l}$) characteristics, as summarized in Table 1. The term $\varepsilon_{ijkl}$ is the robust error term and is clustered by primary sampling units (PSUs), which are the smallest units used by the Nepal Bureau of Statistics to select households for the survey data collection.

Using Eq (1), we test the following hypothesis $H_{0}\;:\alpha =0$ versus $H_{1}\;:\alpha \neq 0$ that the association between maternal employment and child health is not statistically different from zero. We do this in four phases.

First, we estimate the coefficients of maternal employment using the ordinary least squares (OLS) method. If unobserved factors that lead mothers to find employment are also correlated with child nutrition and health, such as financial and non-financial family values, aspirations, and preferences, these OLS estimates are likely to produce biased estimates of the parameters of interest. Simple t-tests of covariates between employed and unemployed mothers within the 2010/11 NLSS in Table 2 confirm that the means of these groups differ significantly across most observable characteristics. The observed differences between employed and unemployed mothers suggest the possibility of selection on unobservable characteristics that cannot be directly controlled for in an OLS framework. Moreover, we could potentially have reverse causality such that mothers of children with poor nutritional status may seek employment to increase household resources and improve their children’s nutrition.

To address the two potential sources of bias, in our second phase, we resort to an instrumental variable (IV) approach. There are a few studies examining maternal employment and child nutrition using an IV approach, but none in the context of Nepal. Diiro, Sam, and Kraybill [8] use the presence of a government employment program in a village as an instrumental variable to estimate the impact of maternal employment on child nutrition in India. However, the NLSS does not offer an IV for the presence of employment opportunities at the national level that we could use. In another study, Jakaria, Bakshi, and Hasan [5] rely on “mothers’ premarital labor-force participation captured by a dichotomous variable whether the mother was employed before marriage” as an instrument for maternal employment in Bangladesh. Again, our dataset does not provide this information. Lastly, Rashad and Sharaf [22] “instrument maternal employment by the cluster average of women’s working status [where clusters are districts in urban areas and villages in rural areas], with the exclusion of the women’s own employment status to avoid in-built association.” This method is more viable in our case, where female employment at the community level should be uncorrelated with unobserved determinants of child health after controlling for observable household, community, and district characteristics. Hence, using the cross-sectional dataset from 2010/11, we construct a community-level female employment rate, which represents the proportion of working females in the district. For each household, this variable is defined as the number of working females in a district in the past 12 months divided by the total number of females in a district, excluding all female household members of the focal household. See for example, Wodon et al. [23] who use a similar identification strategy.

Second, we modify the approach to construct an IV based on deep lag that captures historical communal female employment. Inclusion of this variable in this study is informed by literature that uses previous maternal employment status as instruments [5]. The intuition is that if a mother was employed in the past, she is more likely to be employed in the future; however, her past employment is unlikely to affect the nutritional outcomes of children observed in 2010/11 except through its influence on current employment status. In the case of the NLSS data, because of the large time lag between the two survey waves, it is impossible for maternal employment in 2003/04 to directly influence the health of children born into the 2010/11 cohort because these children had not yet been born. Unfortunately, directly linking children observed in 2010/11 to their mothers in the 2003/04 survey yields a sample that is too small for meaningful statistical analysis. To that end, following Mishra and Sam (2016) [24], we construct the 2010/11 and 2003/04 matched dataset using the coarsened exact matching (CEM) method. CEM is a matching technique that “coarsens” selected variables into groups and divides the sample into several strata, each of which has identical values for all the coarsened pre-treatment covariates [25]. A single treated observation may be matched to multiple comparison observations, and vice versa. The CEM drops all individuals from any stratum that does not match. Here, mothers in the 2010/11 sample are matched with females in the 2003/04 sample on the following variables: gender of the household head, the age and educational attainment of mothers in 2010/11 and females in 2003/04, household size, the number of household members between 16 and 64, and the ratio of females in a household. The goal is to obtain a subset of mothers in 2010/11 that are similar to females in 2003/04. The sample of matched females in 2003/04 do not necessarily have to have children of their own, as long as they are similar on other characteristics to the mothers in 2010/11. Furthermore, the CEM process matches a single observation in 2010/11 (2003/04) to multiple observations in 2003/04 (2010/11), resulting in a one-to-many match. For this reason, rather than generating an IV to capture the previous female employment status on the individual level, we construct a variable that reflects historical female employment on the community level, where community is defined at the primary sampling unit (PSU) as in Wodon et al. [23].

This process results in a total of 114 matched strata (see S1 Appendix, S1 Table for the matching summary of the CEM procedure). Upon merging, we define the lagged community-level female employment status based on the 2003/04 sample by dividing the total female employment in the community in the past seven days by the total number of females residing in the community. This lagged measure of community-level female employment from 2003/04 serves as an instrument for maternal employment in 2010/11. In addition, we control for twenty-three relevant socioeconomic variables, including child characteristics (child’s gender, age, age squared, birth order, vaccination status, and breastfeeding status), mother characteristics (age and education), household characteristics (household head’s gender and ethnicity, age groups of household members, father’s employment, number of adult females in the household, index for hygiene and sanitation, index for female empowerment, household income quintile, distance (in kilometers) to a healthcare facility, primary school, paved road, market, and bank, rural status, and ecological zone), and district characteristics (district-level use of prenatal care), to account for observable factors that could simultaneously influence both the instrument and child nutrition outcomes.

Below we present the first- and second-stages of the IV method:


$$\begin{array}{c} M_{ijkl}=\pi_{0}+\pi_{1}Z_{kl}+\pi_{2}C_{ijkl}+\pi_{3}X_{jkl}+\pi_{4}H_{kl}+\pi_{5}G_{l}+u_{jkl}, \end{array}$$ (2)

where $M_{ijkl}$ indicates maternal employment, $\pi_{1}Z_{kl}$ is a vector of instrument (community-level female employment in 2010/11 and lagged female employment at the community (PSU) level in 2003/04), and $u_{jkl}$ is the error term. The remaining variables are the same as in Eq (1). Eq (2) isolates the exogenous variation in maternal employment driven by past community-level female employment patterns.


$$\begin{array}{c} Y_{ijkl}=\theta_{0}+\theta_{1}{\hat{M}}_{ijkl}+\theta_{2}C_{ijkl}+\theta_{3}X_{jkl}+\theta_{4}H_{kl}+\theta_{5}G_{l}+v_{ijkl}, \end{array}$$ (3)

where ${\hat{M}}_{ijkl}$ indicates predicted maternal employment from the first stage and $\theta_{1}$ is the parameter of interest (causal effect of maternal employment). Standard errors are clustered at the PSU level. To assess the relevance of our instruments, we use the Kleibergen and Paap [26] tests for weak instruments, reporting the Wald F-statistic for the first-stage estimates. Similarly, to assess the validity of our instruments, we use the Hansen J test of overidentifying restrictions and report the associated p-value for the second-stage estimates whenever relevant.

In our third phase, we investigate the heterogeneity of the results by conducting subgroup analyses based on children’s age and gender, rural versus urban residence, ecological region, household income quintile, and whether another adult female (besides the mother) lives in the household. Finally, in the fourth phase, we refine the measurement of maternal employment distinguishing between employment in the agricultural sector, the non-agricultural sector, self-employment, wage employment, and number of hours worked in the past 7 days.

## Results

The results for our analysis are obtained in four stages: (i) an OLS regression, (ii) an IV specification using one instrument (community female employment) and a second IV specification using two instruments (community female employment and lagged community female employment), (iii) subgroup analysis, and (iv) alternative measures of the independent variable—maternal employment. For our primary analysis (i) and (ii), we include two definitions of independent variables: maternal employment in the past 7 days and the past 12 months, followed by only one type of independent variable—maternal employment in the past 12 months for (iii) and (iv).

First, we present results from the OLS regression in Table 3. Columns 1–6 present results for the level of stunting (HAZ), followed by the level of underweight (WAZ), and the level of wasting (WHZ). Specifically, columns 1–3 present results for maternal employment in the past 12 months and columns 4–6 for past 7 days. Based on these six columns for maternal employment in the past 12 months and 7 days, we find no statistically significant association between maternal employment and stunting, underweight, and wasting. In all of these models, we control for a variety of covariates important for child nutrition as discussed in the earlier section.

**Table 3:** OLS results for effects of maternal employment on child nutrition.

	Past 12 months			Past 7 days		
Variables	HAZ	WAZ	WHZ	HAZ	WAZ	WHZ
	(1)	(2)	(3)	(4)	(5)	(6)
Maternal employment	−0.1189	−0.0595	0.0251	−0.0555	−0.0332	0.0063
	(0.0787)	(0.0624)	(0.0676)	(0.0675)	(0.0539)	(0.0562)
Child is female	0.0295	−0.0363	−0.0075	0.0306	−0.0358	−0.0077
	(0.0508)	(0.0442)	(0.0494)	(0.0508)	(0.0442)	(0.0494)
Child age	−0.1170***	−0.0413***	0.0259***	−0.1170***	−0.0412***	0.0259***
	(0.0076)	(0.0057)	(0.0069)	(0.0076)	(0.0057)	(0.0069)
Child age squared	0.0014***	0.0004***	−0.0003***	0.0014***	0.0004***	−0.0003***
	(0.0001)	(0.0001)	(0.0001)	(0.0001)	(0.0001)	(0.0001)
Child birth order	−0.0443	−0.0382	−0.0245	−0.0443	−0.0381	−0.0244
	(0.0305)	(0.0265)	(0.0266)	(0.0304)	(0.0265)	(0.0266)
Ratio of vaccines	0.0116	−0.0984	−0.1026	0.0050	−0.1011	−0.1006
	(0.1379)	(0.0960)	(0.1067)	(0.1371)	(0.0953)	(0.1057)
Breastfeeding status	0.0350	−0.0162	−0.0580	0.0338	−0.0169	−0.0579
	(0.0663)	(0.0528)	(0.0574)	(0.0662)	(0.0526)	(0.0572)
Mother’s age	0.0189**	0.0171***	0.0090	0.0182**	0.0167***	0.0092
	(0.0077)	(0.0062)	(0.0065)	(0.0077)	(0.0062)	(0.0065)
Mother’s education (primary school)	−0.4322***	−0.4266***	−0.2491**	−0.4345***	−0.4280***	−0.2489**
	(0.1270)	(0.1020)	(0.1099)	(0.1271)	(0.1018)	(0.1100)
Mother’s education (middle school)	−0.3189**	−0.2624***	−0.1146	−0.3234***	−0.2647***	−0.1137
	(0.1236)	(0.0953)	(0.0977)	(0.1239)	(0.0955)	(0.0979)
Mother’s education (SLC or above)	−0.3107***	−0.1881**	−0.0148	−0.3126***	−0.1892**	−0.0146
	(0.1144)	(0.0944)	(0.1006)	(0.1142)	(0.0945)	(0.1007)
Father is engaged in wage employment	−0.0887	−0.0721	−0.0307	−0.0861	−0.0709	−0.0314
	(0.0788)	(0.0582)	(0.0651)	(0.0789)	(0.0582)	(0.0650)
Female HH headship	0.0011	0.0943	0.1281*	0.0013	0.0943	0.1281*
	(0.0785)	(0.0591)	(0.0686)	(0.0788)	(0.0592)	(0.0686)
HH head ethnicity (Brahman)	−0.1494	−0.2301**	−0.2200*	−0.1552	−0.2333**	−0.2191*
	(0.1057)	(0.0955)	(0.1162)	(0.1060)	(0.0959)	(0.1164)
HH head ethnicity (Newar)	0.1123	0.3256**	0.3841***	0.1114	0.3255**	0.3846***
	(0.1594)	(0.1313)	(0.1453)	(0.1595)	(0.1311)	(0.1452)
HH head ethnicity (Other)	−0.1704**	−0.0231	0.1111	−0.1665*	−0.0214	0.1101
	(0.0864)	(0.0723)	(0.0836)	(0.0865)	(0.0723)	(0.0835)
Household size	−0.0446	−0.0291	−0.0119	−0.0460	−0.0304	−0.0121
	(0.0450)	(0.0365)	(0.0366)	(0.0447)	(0.0369)	(0.0367)
HH member between 6–15 years	−0.0115	−0.0238	−0.0178	−0.0099	−0.0226	−0.0177
	(0.0598)	(0.0476)	(0.0470)	(0.0596)	(0.0478)	(0.0470)
HH member over 65 years	0.0162	0.0360	0.0348	0.0170	0.0365	0.0347
	(0.0579)	(0.0442)	(0.0496)	(0.0580)	(0.0443)	(0.0495)
Number of other working members	0.0161	0.0340	0.0312	0.0207	0.0371	0.0309
	(0.0304)	(0.0244)	(0.0285)	(0.0309)	(0.0252)	(0.0290)
Number of adult females in HH	0.0799	0.0235	−0.0198	0.0770	0.0224	−0.0188
	(0.0723)	(0.0550)	(0.0569)	(0.0721)	(0.0550)	(0.0570)
Index for hygiene and sanitation	0.2334	0.3201**	0.2690*	0.2586	0.3324**	0.2633*
	(0.1685)	(0.1485)	(0.1548)	(0.1692)	(0.1469)	(0.1537)
Index for female empowerment	0.0577	0.0421	0.0002	0.0617	0.0441	−0.0007
	(0.0417)	(0.0308)	(0.0348)	(0.0416)	(0.0308)	(0.0348)
Distance to healthcare facility	−0.0281***	−0.0100	0.0112	−0.0293***	−0.0106	0.0115
	(0.0105)	(0.0080)	(0.0087)	(0.0104)	(0.0080)	(0.0087)
Rural	−0.1207	−0.0443	0.0459	−0.1313	−0.0493	0.0484
	(0.0892)	(0.0722)	(0.0756)	(0.0883)	(0.0722)	(0.0760)
Hill region	−0.0080	−0.0622	−0.0933	−0.0100	−0.0642	−0.0938
	(0.1321)	(0.1117)	(0.1214)	(0.1338)	(0.1126)	(0.1220)
Tarai region	0.1402	−0.2493**	−0.4849***	0.1491	−0.2467**	−0.4887***
	(0.1411)	(0.1171)	(0.1252)	(0.1431)	(0.1185)	(0.1262)
District-level use of prenatal care	−1.6800**	−2.1922***	−1.7729***	−1.6249**	−2.1667***	−1.7866***
	(0.7885)	(0.6572)	(0.6349)	(0.7905)	(0.6537)	(0.6311)
HH in 2nd income quintile	0.2125**	0.1042	−0.0424	0.2149**	0.1056	−0.0426
	(0.0965)	(0.0770)	(0.0829)	(0.0969)	(0.0770)	(0.0832)
HH in 3rd income quintile	0.2502**	0.1400*	0.0064	0.2534***	0.1421*	0.0062
	(0.0968)	(0.0741)	(0.0813)	(0.0978)	(0.0746)	(0.0817)
HH in 4th income quintile	0.2013**	0.0937	−0.0203	0.2099**	0.0984	−0.0217
	(0.0972)	(0.0798)	(0.0891)	(0.0984)	(0.0802)	(0.0893)
HH in 5th income quintile	0.3012***	0.2108**	0.0632	0.3081***	0.2144**	0.0619
	(0.1104)	(0.0897)	(0.1008)	(0.1113)	(0.0900)	(0.1008)
Constant	0.0293	−0.6079**	−0.8440***	−0.0201	−0.6290**	−0.8300***
	(0.3511)	(0.3020)	(0.3052)	(0.3535)	(0.3028)	(0.3019)
Observations	2,427	2,427	2,427	2,427	2,427	2,427
R-squared	0.2813	0.2035	0.1060	0.2808	0.2033	0.1060

Notes: ***p < 0.01, **p < 0.05, *p < 0.1; robust standard errors in parentheses; number of vaccines includes BCG, polio, DPT, measles, and Hp-B; controlling for asset class instead of income quintiles produces qualitatively similar results.

Second, we present results from the IV regression (with community level IV) in Table 4. Similar to Table 3, the first column presents results for HAZ, followed by the second column with results for WAZ, and the third column with results for WHZ. For maternal employment in the past week, the first-stage F-test suggests that the instrument is relevant, with a Wald F-statistic of 27.9. We find that the coefficient on maternal employment is 0.024 for stunting (Column 1), 0.27 for underweight (Column 2), and 0.29 for wasting (Column 3), although none of them is statistically significant. We observe similar results for the alternative measure of maternal employment, measured as employment in the past 12 months, in Columns 4–6. The instrumental variable appears relevant for the alternative measure of maternal employment, as indicated by a first-stage Wald F-statistic of 111.5. These results imply that maternal employment does not have any statistically significant impact on child nutrition. Even though the results are statistically insignificant, comparing the coefficient magnitudes across the OLS and IV regressions points to the possibility of downward bias because the IV coefficient estimates are larger in magnitude than the corresponding OLS estimates.

**Table 4:** Second-stage 2SLS regression results with instrumental variable: community female employment.

	Past 7 days^a^			Past 12 months^b^		
	HAZ	WAZ	WHZ	HAZ	WAZ	WHZ
Mother is employed	0.0243	0.2735	0.2984	0.0149	0.1675	0.1828
	(0.4391)	(0.3618)	(0.3569)	(0.2692)	(0.2224)	(0.2186)
Constant	0.5235	−0.2228	−0.6969*	0.5259	−0.1956	−0.6672*
	(0.4033)	(0.3529)	(0.3656)	(0.3815)	(0.3283)	(0.3420)
Observations	2,378	2,378	2,378	2,378	2,378	2,378

Notes: Clustered robust standard errors (at PSU level) in parentheses. Statistical significance: ***p < 0.01, **p < 0.05, *p < 0.1. Control variables include child characteristics (child’s gender, age, age squared, birth order, vaccination status, and breastfeeding status), mother characteristics (age and education), household characteristics (household head’s gender and ethnicity, age groups of household members, father’s employment, number of adult females in the household, index for hygiene and sanitation, index for female empowerment, household income quintile, distance to a healthcare facility, primary school, paved road, market, and bank, rural status, and ecological zone), and district characteristics (district-level use of prenatal care).

^a^First stage weak identification test: Kleibergen–Paap Wald rk F-statistic: 27.95.

^b^First stage weak identification test: Kleibergen–Paap Wald rk F-statistic: 111.50.

Next, we present results from the IV regression with two IVs in Table 5. Parallel to earlier tables, the results for stunting, underweight, and wasting are presented in the first, second, and third columns, respectively. For maternal employment in the past 7 days, the first-stage Wald F-statistic (19.4) suggests that the instruments are relevant, while the Hansen J-stat (p-value = 0.80) fails to reject the validity of the overidentifying restrictions. We find that the coefficient on maternal employment is −0.01 for HAZ (Column 1), 0.21 for WAZ (Column 2), and 0.21 for WHZ (Column 3), and none of the coefficients is statistically significant. The results are similar when we measure maternal employment as employment in the past 12 months (Columns 4–6).The instruments appear relevant for the alternative measure of maternal employment (Wald F-statistic = 56.7), and the Hansen J-stat (*p* = 0.80) does not reject the validity of the overidentifying restrictions. The lack of statistical significance for these coefficients reinforces earlier findings from Table 4 that maternal employment has no statistically significant association with child nutrition and growth in Nepal.

**Table 5:** Second-stage 2SLS regression results with instrumental variables: community female employment (past 12 months) and lagged community female employment (past 7 days).

	Past 7 days^a^			Past 12 months^b^		
	HAZ	WAZ	WHZ	HAZ	WAZ	WHZ
Mother is employed	−0.0082	0.2084	0.2130	0.0130	0.1652	0.1793
	(0.4077)	(0.3342)	(0.3289)	(0.2686)	(0.2220)	(0.2178)
Constant	0.5391	−0.1915	−0.6559*	0.5270	−0.1941	−0.6651*
	(0.3937)	(0.3445)	(0.3611)	(0.3812)	(0.3283)	(0.3423)
Observations	2,378	2,378	2,378	2,378	2,378	2,378

Notes: Clustered robust standard errors (at PSU level) in parentheses. Statistical significance: ***p < 0.01, **p < 0.05, *p < 0.1. Control variables include child characteristics (child’s gender, age, age squared, birth order, vaccination status, and breastfeeding status), mother characteristics (age and education), household characteristics (household head’s gender and ethnicity, age groups of household members, father’s employment, number of adult females in the household, index for hygiene and sanitation, index for female empowerment, household income quintile, distance to a healthcare facility, primary school, paved road, market, and bank, rural status, and ecological zone), and district characteristics (district-level use of prenatal care).

^a^First stage weak identification test: Kleibergen–Paap Wald rk F-statistic: 19.4; Hansen’s J-statistic p-value: 0.81.

^b^First stage weak identification test: Kleibergen–Paap Wald rk F-statistic: 56.7; Hansen’s J-statistic p-value: 0.82.

Third, we present OLS results from subsample analyses by child age (infants/toddlers versus preschoolers), child gender, rural-urban residence, ecological zone (Hill versus Tarai), household income quintile (bottom two versus top two quintiles), and the presence of another adult female in the household. These results are reported in S2–S7 Tables (see S1 Appendix for details). We report OLS estimates for the subsample analyses because the instruments do not exhibit sufficient strength, limiting the credibility of IV estimation in these cases. The primary determinant variable used is maternal employment in past 12 months. Consistent with the previous tables, Columns 1–3 present results for HAZ, WAZ, and WHZ, respectively. Overall, the estimates are not statistically significant across age groups, gender, rural-urban residence, ecological regions, and income quintiles with a few exceptions. These exceptions are: (i) a negative and marginally statistically significant association (at the 10% level) for HAZ among infants/toddlers and for those in the Hill region (S2 and S5 Tables, S1 Appendix), and (ii) a statistically significant negative association with both HAZ and WAZ in households with no other adult females present (S7 Table, S1 Appendix). Finally, we present results from IV regression by types of employment, including agriculture and non-agricultural sector (S8 Table, S1 Appendix), self-employment and wage employment (S9 Table, S1 Appendix), and number of hours worked in past 7 days (S10 Table, S1 Appendix). The estimated coefficients remain statistically insignificant across all three outcomes. The Kleibergen-Paap Wald rk F-statistics for the non-agricultural sector, wage-employment, and number of hours worked suggest that the design cannot credibly identify effects for these employment categories.

Overall, our results fall in the middle of the spectrum of findings in the literature on maternal employment and child nutrition and are consistent with the findings of Baker and Milligan [12] who find no effect on most child health indicators. One set of studies have found that, due to added burden of outside employment and reduced time for caregiving, maternal employment can translate into negative impacts on children’s nutrition and health [2,9–11]. Alternatively, another set of studies have found that, through added earnings, maternal employment may improve child health and nutrition outcomes [6–8]. We believe that there could be a few context-specific reasons why we do not find any statistically significant impact of maternal employment on child health in Nepali. First, in our sample, over 63% of mothers are employed in the agriculture sector and 43% are self-employed. This suggests that much of maternal employment is concentrated in small-scale agricultural and self-employment activities, which are often informal and characterized by low earnings. As a result, maternal employment may not generate sufficient income to substantially improve household resources or child health outcomes. In fact, only about 9% of mothers are engaged in wage employment. Second, there might be a time versus income tradeoff since agricultural labor is time-intensive and physically demanding which may reduce a mother’s ability to provide care for their children, potentially offsetting some or all of the benefits of additional earnings [27]. Third, patriarchal gender norms in Nepal may limit mothers’ control over how their earnings are used, as financial decisions often rest with husbands and senior family members, particularly mothers-in-law [15]. As a result, child nutrition may depend more on women’s bargaining power and control over resources than on labor force participation itself [28,29]. Moreover, because agricultural households are often organized as joint families, childcare decisions are frequently influenced by older women. This pattern is also reflected in our sample, where approximately 60% of households include at least one additional adult female. Although maternal employment may be socially accepted, the lack of formal childcare services means that caregiving is often provided by grandparents or older siblings, potentially leading to inconsistencies in feeding, hygiene, and other childcare practices [30]. These offsetting mechanisms, income, caregiving, and intrahousehold bargaining, may help explain why we find no statistically significant effects of maternal employment on children’s anthropometric outcomes.

## Conclusion

Developing countries face several barriers to poverty reduction. One of these barriers is the high prevalence of undernutrition in children which leads to lower educational attainment and reduced lifetime earnings. Therefore, addressing the factors that can impact child nutrition is an important issue. In this regard, we investigate if maternal employment boosts child growth and nutritional status, quantified by height-for-age, weight-for-age, and weight-for-height z-scores for children under the age of five, using a national-level panel dataset from Nepal. In doing so, we contribute to the existing literature by adding evidence from a developing country in the South Asian context and using robust econometric techniques.

There are two schools of thought on how maternal employment can impact child nutrition. One states that employed mothers can use their earnings to invest more in children’s health and nutrition, leading to positive child outcomes. The other argues that employed mothers may have less time for childcare because they must balance caregiving, household responsibilities, and paid work. We employed two econometric techniques and various specifications, including subgroup analysis, to establish the relationship between maternal employment and child nutrition. Our results indicate that maternal employment has no statistically significant impact on children’s nutrition, measured by stunting, underweight, and wasting.

The insignificant relationship between maternal employment and child nutrition in Nepal likely reflects offsetting mechanisms documented in the literature [12,14]. Working mothers are mostly engaged in the informal sector and self-employment which may not meaningfully increase household income. Alternatively, even when employment may raise household income, it may simultaneously reduce the time available for childcare and feeding, particularly in informal, low-productivity jobs common in Nepal and South Asia. Moreover, employment alone may not translate into better child outcomes without women’s control over household resources and decision-making, which remain constrained in patriarchal and gerontocratic settings [15]. Finally, broader factors such as maternal education and sanitation may play a more dominant role in shaping child nutrition outcomes in Nepal. Hence, there is a need for policies that support women’s labor force participation and access to formal childcare services.

## Supporting information

S1 Appendix Supporting information. Maternal employment and child health.
(PDF)
