# Supplementary material for: Impact of maternal employment on child health and nutrition: Evidence from Nepal
Source: PLoS One. 2026 Aug 13;21(8):e0355481. doi: 10.1371/journal.pone.0355481 (PMC13472435; doi:10.1371/journal.pone.0355481)
Supplement: Supplementary file 1 [file pone.0355481.s001.pdf]

Impact of maternal employment on child health and nutrition: evidence from Nepal

Khushbu Mishra[1*], Olga Kondratjeva[2], Abdoul G. Sam[3**]

1 Department of Economics, Stetson University, Deland, Florida, United States of America
2 Washington State Employment Security Department, Olympia, Washington, United States of America
3 Department of Agricultural, Environmental and Development Economics, The Ohio State University, Columbus, Ohio, United States of America

[*]Corresponding author email: kmishra@stetson.edu
[**]Corresponding author email: sam.7@osu.edu

Supporting information

Table S1. Matching summary of the coarsened exact matching (CEM) procedure.

Number of strata: 261. Number of matched strata: 114.

Matching summary

	2003/04	2010/11
All	12,036	2,431
Matched	11,052	2,410
Unmatched	984	21

Univariate imbalance

	L1	mean	min	25%	50%	75%	max
Mother's age	0.23	−0.68	9.00	0.00	0.00	0.00	−47.00
Mother's education	0.00	0.00	0.00	0.00	0.00	0.00	0.00
Female household headship	0.00	0.00	0.00	0.00	0.00	0.00	0.00
Household size	0.05	0.12	1.00	0.00	0.00	0.00	−1.00
No. of household members 16–64 years old	0.10	−0.32	1.00	0.00	0.00	−1.00	−2.00
Household female ratio	0.07	−0.01	0.10	−0.02	0.00	0.00	−0.20

Table S2. OLS regression results by child age.

	Infants/Toddlers			Preschoolers		
**Variables**	**HAZ**	**WAZ**	**WHZ**	**HAZ**	**WAZ**	**WHZ**
Mother employed	−0.1884*	−0.0447	0.1183	−0.0683	−0.0875	−0.0758
	(0.1057)	(0.0821)	(0.0922)	(0.1056)	(0.0845)	(0.0937)
Constant	1.0721**	0.1972	−0.6213	−2.1743***	−1.3755***	−0.1307
	(0.4945)	(0.3993)	(0.3948)	(0.4457)	(0.3702)	(0.4429)
Observations	1,382	1,382	1,382	996	996	996

Notes: Mother employed is a binary indicator for those who are employed in the past 12 months; clustered robust standard errors (at PSU level) in parentheses. Statistical significance: $^{***}p < 0.01$, $^{**}p < 0.05$, $^{*}p < 0.1$. Controls: child characteristics (gender, birth order, vaccination status, breastfeeding status), mother characteristics (age, education), household characteristics (household head's gender and ethnicity, age groups of household members, father's employment, number of household females, index for hygiene and sanitation, index for female empowerment, household income quintile, distance to a healthcare facility, primary school, paved road, market, and bank, rural status, and ecological zone), and district characteristics (district-level use of prenatal care).

Table S3. OLS regression results by child gender.

	Girls			Boys		
**Variables**	**HAZ**	**WAZ**	**WHZ**	**HAZ**	**WAZ**	**WHZ**
Mother employed	−0.0723	−0.0116	0.0518	−0.1668	−0.1020	0.0104
	(0.1036)	(0.0935)	(0.0934)	(0.1074)	(0.0771)	(0.0938)
Constant	0.1212	−0.2287	−0.3186	1.0908**	0.1625	−0.6860*
	(0.4624)	(0.4105)	(0.4300)	(0.4262)	(0.3325)	(0.3754)
Observations	1,145	1,145	1,145	1,233	1,233	1,233

Notes: Mother employed is a binary indicator for those who are employed in the past 12 months; clustered robust standard errors (at PSU level) in parentheses. Statistical significance: $^{***}p < 0.01$, $^{**}p < 0.05$, $^{*}p < 0.1$. Controls: child characteristics (age, age squared, birth order, vaccination status, breastfeeding status), mother characteristics, household characteristics(household head's gender and ethnicity, age groups of household members, father's employment, number of household females, index for hygiene and sanitation, index for female empowerment, household income quintile, distance to a healthcare facility, primary school, paved road, market, and bank, rural status, and ecological zone), and district characteristics (district-level use of prenatal care).

Table S4. OLS regression results by rural-urban location.

	Rural			Urban		
**Variables**	**HAZ**	**WAZ**	**WHZ**	**HAZ**	**WAZ**	**WHZ**
Mother employed	−0.1175	−0.0089	0.1049	−0.1002	−0.0906	−0.0312
	(0.1024)	(0.0755)	(0.0848)	(0.1255)	(0.1109)	(0.1200)
Constant	0.3249	−0.0801	−0.3547	1.1037*	−0.1043	−1.2045**
	(0.3705)	(0.3108)	(0.3439)	(0.6470)	(0.5543)	(0.5748)
Observations	1,810	1,810	1,810	568	568	568

Notes: Clustered robust standard errors (at PSU level) in parentheses. Statistical significance: ***$p < 0.01$, **$p < 0.05$, *$p < 0.1$. Control variables include child characteristics (child's gender, age, age squared, birth order, vaccination status, and breastfeeding status), mother characteristics (age and education), household characteristics (household head's gender and ethnicity, age groups of household members, father's employment, number of household females, index for hygiene and sanitation, index for female empowerment, household income quintile, distance to a healthcare facility, primary school, paved road, market, and bank, and ecological zone), and district characteristics (district-level use of prenatal care).

Table S5. OLS regression results by ecological region.

	Hill			Tarai		
**Variables**	**HAZ**	**WAZ**	**WHZ**	**HAZ**	**WAZ**	**WHZ**
Mother employed	−0.2420*	−0.1691	0.0012	−0.0386	−0.0240	−0.0112
	(0.1399)	(0.1195)	(0.1262)	(0.1009)	(0.0769)	(0.0881)
Constant	0.1061	−0.3230	−0.5448	1.2822***	0.2178	−0.7065*
	(0.4820)	(0.3726)	(0.4047)	(0.4418)	(0.3650)	(0.4121)
Observations	1,153	1,153	1,153	1,035	1,035	1,035

Notes: Clustered robust standard errors (at PSU level) in parentheses. Statistical significance: ***$p < 0.01$, **$p < 0.05$, *$p < 0.1$. Control variables include child characteristics (child's gender, age, age squared, birth order, vaccination status, and breastfeeding status), mother characteristics (age and education), household characteristics (household head's gender and ethnicity, age groups of household members, father's employment, number of household females, index for hygiene and sanitation, index for female empowerment, access to a healthcare facility, primary school, paved road, market, and bank, and rural status), and district characteristics (district-level use of prenatal care).

Table S6. OLS regression results by wealth quintile.

	1st and 2nd quintile			4th and 5th quintile		
**Variables**	**HAZ**	**WAZ**	**WHZ**	**HAZ**	**WAZ**	**WHZ**
Mother employed	−0.1230	−0.0587	0.0361	−0.1320	−0.0887	−0.0108
	(0.1272)	(0.1075)	(0.1393)	(0.1045)	(0.0885)	(0.0921)
Constant	−0.2802	−0.5101	−0.4371	1.4316***	0.4448	−0.5623
	(0.5341)	(0.4654)	(0.5117)	(0.5273)	(0.4247)	(0.4430)
Observations	952	952	952	947	947	947

Notes: Clustered robust standard errors (at PSU level) in parentheses. Statistical significance: ***$p < 0.01$, **$p < 0.05$, *$p < 0.1$.Control variables include child characteristics (child's gender, age, age squared, birth order, vaccination status, and breastfeeding status), mother characteristics (age and education), household characteristics (household head's gender and ethnicity, age groups of household members, father's employment, number of household females, index for hygiene and sanitation, index for female empowerment, access to a healthcare facility, primary school, paved road, market, and bank, rural status, and ecological zone), and district characteristics (district-level use of prenatal care).

Table S7. OLS regression results by adult females' presence in the household.

	No adult females			One or more adult females		
**Variables**	**HAZ**	**WAZ**	**WHZ**	**HAZ**	**WAZ**	**WHZ**
Mother employed	−0.2808**	−0.2853***	−0.1684	−0.0456	0.0637	0.1457*
	(0.1254)	(0.0999)	(0.1218)	(0.0984)	(0.0755)	(0.0815)
Constant	0.7485	−0.4294	−1.2418**	0.6383	0.2035	−0.2093
	(0.5182)	(0.4206)	(0.4930)	(0.4637)	(0.3659)	(0.3919)
Observations	1,005	1,005	1,005	1,373	1,373	1,373

Notes: Clustered robust standard errors (at PSU level) in parentheses. Statistical significance: ***$p < 0.01$, **$p < 0.05$, *$p < 0.1$. Control variables include child characteristics (child's gender, age, age squared, birth order, vaccination status, and breastfeeding status), mother characteristics (age and education), household characteristics (household head's gender and ethnicity, age groups of household members, father's employment, index for hygiene and sanitation, index for female empowerment, distance to a healthcare facility, primary school, paved road, market, and bank, rural status, and ecological zone), and district characteristics (district-level use of prenatal care).

Table S8. IV regression results by employment in agricultural and non-agricultural sector.

	Agriculture[a]			Non-agriculture[b]		
**Variables**	**HAZ**	**WAZ**	**WHZ**	**HAZ**	**WAZ**	**WHZ**
Mother employed	0.0104	0.1497	0.1618	0.0069	0.7745	0.8118
	(0.2463)	(0.2043)	(0.2003)	(1.3951)	(1.1639)	(1.1360)
Constant	0.5310	−0.1499	−0.6167*	0.5328	−0.3472	−0.8217
	(0.3548)	(0.3013)	(0.3166)	(0.5755)	(0.5242)	(0.5278)
Observations	2,378	2,378	2,378	2,378	2,378	2,378

Notes: Mother employed is a binary for those who are employed in the past 12 months; instrumental variables: community female employment (past 12 months) + lagged community female employment (past 7 days); clustered robust standard errors (at PSU level) in parentheses. Statistical significance: ***$p < 0.01$, **$p < 0.05$, *$p < 0.1$. Control variables include child characteristics (child's gender, age, age squared, birth order, vaccination status, and breastfeeding status), mother characteristics (age and education), household characteristics (household head's gender and ethnicity, age groups of household members, father's employment, number of household females, index for hygiene and sanitation, index for female empowerment, distance to a healthcare facility, primary school, paved road, market, and bank, rural status, and ecological zone), and district characteristics (district-level use of prenatal care).
[a] First stage weak identification test: Kleibergen–Paap Wald rk F-statistic: 67.2; J-statistic p-value: 0.81.
[b] First stage weak identification test: Kleibergen–Paap Wald rk F-statistic: 2.35.

Table S9. IV regression results by self- and wage employment.

	Self-employment[a]			Wage employment[b]		
**Variables**	**HAZ**	**WAZ**	**WHZ**	**HAZ**	**WAZ**	**WHZ**
Mother employed	0.0121	0.1411	0.1538	−0.7113	0.5606	0.2029
	(0.2276)	(0.1889)	(0.1853)	(4.5037)	(3.5520)	(3.8565)
Constant	0.5277	−0.1778	−0.6477**	0.6998	−0.2211	−0.6004
	(0.3695)	(0.3155)	(0.3302)	(1.0914)	(0.8993)	(0.9861)
Observations	2,378	2,378	2,378	2,378	2,378	2,378

Notes: Self and wage employment are for past 12 months; clustered robust standard errors (at PSU level) in parentheses. Statistical significance: ***$p < 0.01$, **$p < 0.05$, *$p < 0.1$. Control variables include child characteristics (child's gender, age, age squared, birth order, vaccination status, and breastfeeding status), mother characteristics (age and education), household characteristics (household head's gender and ethnicity, age groups of household members, father's employment, index for hygiene and sanitation, index for female empowerment, distance to a healthcare facility, primary school, paved road, market, and bank, rural status, and ecological zone), and district characteristics (district-level use of prenatal care).
[a] First stage weak identification test: Kleibergen–Paap Wald rk F-statistic: 70.2; Hansen's J-statistic p-value: 0.82.
[b] First stage weak identification test: Kleibergen–Paap Wald rk F-statistic: 0.34.

Table S10. IV regression results by number of hours worked in the past 7 days.

Variables	HAZ	WAZ	WHZ
Hours worked	0.0235	−0.0287	0.0092
	(0.0514)	(0.0450)	(0.0500)
Constant	0.5307	−0.0995	−0.5641*
	(0.3433)	(0.2859)	(0.3009)
Observations	2,378	2,378	2,378

Notes: Clustered robust standard errors (at PSU level) in parentheses. Statistical significance: $^{***}p < 0.01$, $^{**}p < 0.05$, $^{*}p < 0.1$. Control variables include child characteristics (child's gender, age, age squared, birth order, vaccination status, and breastfeeding status), mother characteristics (age and education), household characteristics (household head's gender and ethnicity, age groups of household members, father's employment, index for hygiene and sanitation, index for female empowerment, distance to a healthcare facility, primary school, paved road, market, and bank, rural status, and ecological zone), and district characteristics (district-level use of prenatal care).
First stage weak identification test: Kleibergen–Paap Wald rk F-statistic: 4.35.
